# Anti-stress activity of hydro-alcoholic extract of *Eugenia caryophyllus* buds (clove)

**DOI:** 10.4103/0253-7613.48889

**Published:** 2009-02

**Authors:** Anand Kumar Singh, Sunil S. Dhamanigi, Mohammed Asad

**Affiliations:** Krupanidhi College of Pharmacy, # 5, Sarjapur Road, Koramongala, Bangalore, India

**Keywords:** Anoxic stress, anti-stress, cold restraint, eugenia caryophyllus, sound stress

## Abstract

**Objective:**

The present study was undertaken to evaluate the anti-stress effect of the hydro-alcoholic extract of clove.

**Methodology:**

The anti-stress effect was evaluated on cold restraint induced gastric ulcers, sound stress induced biochemical changes and anoxic stress induced convulsions. Clove extract was administered orally at two different doses of 100 and 200 mg/kg. Zeetress, a known anti-stress formulation (14 mg/kg *p.o*) was used as the standard drug.

**Results:**

Both the doses of clove extract showed good anti-stress effect in all the tested models. The clove extract reduced the development of cold restraint induced gastric ulcers and prevented the biochemical changes induced by sound stress such as increase in plasma levels of aspartate aminotransferase, alanine aminotransferase, alkaline phosphatase, glucose, cholesterol and corticosterone. Clove extract was also effective in increasing the latency of anoxic stress induced convulsions in mice.

**Conclusion:**

The hydro-alcoholic extract of clove at doses of 100 and 200 mg/kg orally possesses good anti-stress activity.

## Introduction

Stress is a common phenomenon that is experienced by every individual. When stress becomes extreme, it is harmful for the body and, hence, needs to be treated. Stress is involved in the pathogenesis of a variety of diseases that includes psychiatric disorders such as depression and anxiety, immunosuppression, endocrine disorders including diabetes mellitus, male impotence, cognitive dysfunction, peptic ulcer, hypertension and ulcerative colitis.[1]

*Eugenia caryophyllus* (clove), belonging to the family Myrtaceae, has a number of medicinal properties and its systemic as well as local use has been advocated in traditional medicine. Clove is reported to possess anti-oxidant,[2] anti-bacterial,[3] anti-pyretic,[4] anti-candidal,[5] local anesthetic[6] and aphrodisiac[7] activities. It is widely used as an aromatic stimulant, antispasmodic and carminative spice. Clove contains 14-20% of volatile oil that includes eugenol, acetyleugenol, sesquiterpenes (α-and β-caryophyllenes) and small quantities of esters, ketones and alcohol. Clove also contains tannins, sitosterol and stigmosterol.[8]

Clove has been reported to possess a potent anti-oxidant activity *in vitro*,[9] which reduces the oxidative stress in the body.[10] Since *Eugenia caryophyllus* has a number of medicinal properties and is a potent anti-oxidant, the present study was undertaken to evaluate its anti-stress effect in experimental animals. The anti-stress effect of clove was compared with zeetress, a polyherbal formulation containing three known anti-stress herbs, *Ocimum sanctum, Withania somnifera* and *Emblica officinalis*. Earlier studies carried out with both the formulation and its constituents showed that they possess very good anti-stress and anti-oxidant effect.[11–17] The formulation was selected as it is easily available and ready to use.

## Materials and Methods

### Experimental animals

Albino Wistar rats weighing between 175-250 gm and Swiss albino mice weighing 25-40 gm of either sex were used. The experimental animals were maintained under 12:12 h light dark cycle, in an animal house with controlled temperature. The Institutional Animal Ethics Committee approved the experimental protocol.

### Preparation of the extract

Clove was extracted using 70% v/v ethanol in a Soxhlet apparatus (Borosil, Mumbai, India). The extract obtained was dried using a rotavapor (Roteva-Equitron, Medica Instruments, Mumbai, India). The yield was 49% w/w. The extract was subjected to preliminary qualitative phytochemical analysis.

### Acute oral toxicity study

The acute oral toxicity was determined in mice, according to the OPPTS (Office of Prevention, Pesticide and Toxic Substance) guidelines, following limit test procedure.[18] The extract was suspended using 0.5% sodium carboxy methylcellulose, and was administered orally. The concentration was adjusted in such a way that it did not exceed 1 ml/100g of the mouse.

### Selection of dose and treatment period

The treatment period consisted of 14 days in all the models, except cold restraint induced gastric ulcers. The following doses of drugs were administered:

Group - I - Vehicle (1 ml /100 gm *p.o* in mice and 5 ml/ kg *p.o* in rats)

Group - II - Zeetress (14 mg/kg *p.o*)

Group III and Group IV: Clove extract (100 mg/kg *p.o* or 200 mg/kg *p.o*)

### Screening for anti-stress activity

1. Cold restraint stress induced gastric ulcers:[19,20] The clove extract was administered 30 min prior to stress. Female albino rats were placed in a restraint cage and the cage was placed at a temperature of 2°C for three hours. The rats were then sacrificed with an overdose of ether anesthesia and the stomach was isolated and cut open along the greater curvature. The stomach samples were scanned using a computer scanner and the total mucosal area and total ulcerated area were measured using a public domain image processing and analysis program developed at the National Institute of Health, USA. The PC version of the program was downloaded free from Scion (http://www.scionocrp.com) (Scion Image for Windows, Release Beta 4.0.2). The scale was set at 6.1 pixels per millimeter. The ulcer index was determined using the following formula:[21]
  Ulcer index = 10/X
  Where X = Total mucosal area / Total ulcerated area.
2. Sound stress induced biochemical changes:[22] The apparatus used for induction of sound/noise stress was a soundproof closed wooden chamber. A continuous sound was generated using a radio frequency tuner to induce sound stress.
  Male albino rats were pretreated with drugs, once a day for 14 days. On the 14^th^ day, one hour after the drug administration, the animals were exposed to sound stress by placing them in the audiogenic test chamber for 30 min individually. The animals were then removed from the chamber and blood was collected. The plasma was used for the estimation of AST (aspartate aminotransferase), ALT (alanine aminotransferase), ALP (alkaline phosphatase), glucose, total cholesterol, triglycerides and corticosterone levels.[22–27]
3. Anoxic stress tolerance:[28] Conical flasks of 250 ml capacity were used for the study. The flasks were made airtight using rubber cork, before the start of the experiment. Male albino mice weighing 25 to 34 g were treated with drug/vehicle for 14 days. Each animal was then kept in the airtight vessel and the time taken for the first convulsion was noted. The animal was removed immediately from the vessel and resuscitated if needed.

### Statistical analysis

All values are expressed as mean±SD. Statistical significance was determined using one way ANOVA, followed by Dunnett's test. *P*<0.05 was considered to be significant.

## Results

Preliminary phytochemical analysis of the extract revealed the presence of alkaloids, carbohydrates, steroids, tannins and flavanoids.

In acute oral toxicity study, the clove extract was safe at a dose of 2000 mg/kg and 1/10^th^ and 1/20^th^ of this dose was used for evaluation of anti-stress effect.

**Figure 1:**
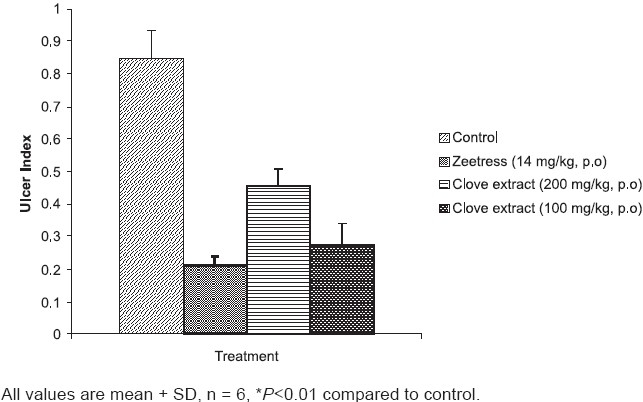
Effect of clove extract and zeetress on cold restraint stress induced gastric ulcers in rats

Administration of zeetress and both the doses of clove extract significantly reduced the development of gastric ulcers induced by cold restraint stress [Figure 1].

**Figure 2:**
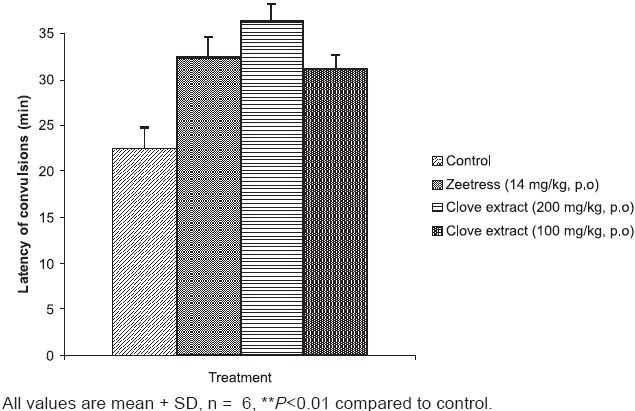
Effect of clove extract and zeetress on anoxic stress tolerance in mice

The induction of sound stress led to a rise in plasma ALT, ALP, glucose, cholesterol and corticosterone levels. All the three treatments produced a significant reduction in ALT levels (*P*<0.01), as compared to controls. However, no significant effect was observed on the AST levels, except at the high dose of clove extract (200 mg/kg, p.o.). The plasma glucose was significantly increased, when the animals were subjected to sound stress compared to control (*P*<0.01). Pretreatment of animals with the zeetress or the high dose of clove extract prevented this (*P*<0.01). All the three treatments prevented the increase in plasma ALP, cholesterol and corticosterone levels. The plasma triglyceride level was, however, reduced after the animals were subjected to sound stress. Treatment of animals with different drugs, before subjecting them to sound stress, prevented the decrease in plasma triglyceride levels [Table 1]. Pretreatment of the animals with zeetress and low as well as high dose of clove extract significantly increased the latency of convulsions in anoxic stress test [Figure 2].

**Table 1:** Effect of clove extract and zeetress on sound stress induced biochemical changes in rats

Treatment group	ALT (U/L)	AST (U/L)	Plasma glucose mg/dl	Plasma Alkaline phosphatase (U/L)	Plasma cholesterol (mg/dl)	Plasma triglycerides (mg/dl)	Plasma corticosterone (μg/dl)
Control	37.95 ± 4.97	96.50 ± 27.41	117.00 ± 17.68	415.67 ± 11.34	92.52 ± 7.23	118.33 ± 8.79	22.18 ± 2.84
Sound stress	61.73 ± 8.81++	118.91± 10.21	143.53 ± 17.68++	449.40 ± 35.35+	135.52 ± 10.53++	85.25 ± 6.81++	46.98 ± 4.73++
Zeetress (14 mg/kg *p.o*)	24.71 ± 2.67**	120.36 ± 14.79	86.33 ± 25.81**	401.75 ± 4.95**	105.00 ± 9.08**	120.20 ± 4.68**	27.98 ± 2.03**
Clove extract (100 mg/kg *p.o*)	31.01 ± 0.44**	110.06 ± 9.38	125.93 ± 14.30	393.95 ± 21.31**	98.33 ± 7.64**	110.00 ± 5.46*	34.89 ± 1.25*
Clove extract (200 mg/kg *p.o*)	25.71 ± 3.30**	99.41 ± 9.82**	60.83 ± 20.40**	379.95 ± 13.74**	110.00 ± 7.76**	122.22 ± 5.36**	29.40 ± 1.27**

Values are mean ± SD

+ *P*<0.05

++ *P*< 0.01, compared to control

* *P*<0.05

** *P*<0.01 compared to sound stress control

## Discussion

The results of the present study show that the hydro-alcoholic extract of clove possesses significant anti-stress activity. The high dose of clove extract prevented the development of gastric ulcers in cold restraint stress induced gastric ulcer model and decreased the levels of biochemical markers of cell damage. An increased latency of anoxic stress induced convulsions was also observed with clove extract.

The cold restraint gastric ulcer model is used to evaluate agents that can inhibit the development of gastric ulcers by their anti-ulcer and/or anti-stress effect.[20] Clove is reported to possess gastric anti-ulcer effect against ethanol induced gastric ulcers due to its cytoprotective action.[29] Hence, it is possible that the reduction of gastric ulcers by clove extract may be due to its anti-stress as well as cytoprotective effect.

The sound stress induced an increase in plasma AST, ALT, ALP, cholesterol and cortisterone levels and a decrease in the triglycerides levels. These alterations are due to the stimulation of hypothalamo-pituitary axis (HPA) and sympathetic nervous system resulting in liberation of catecholamines and glucocorticoids.[30] This causes mobilization of lipids and an enhanced synthesis of cholesterol.[22] Triglycerides, unlike cholesterol, recorded a decrease because triglycerides act as a rapid source of energy during stress conditions. The preventive effect of clove on the sound stress induced biochemical changes indicates its anti-stress activity. The effect of clove may be due to its effect on the central nervous system or endocrines and it may also be due to its antioxidant effect as anti-oxidants are known to prevent stress induced damage due to generation of free radicals.[10]

The last model followed for confirming the anti-stress activity of clove extract was anoxic stress induced convulsions. In this method too, both doses of clove showed significant effect. The high dose of the clove extract (200 mg/kg, *p.o*) was more effective than the lower dose (100 mg/kg, *p.o*) in reducing the sound stress induced biochemical changes while no such dose dependent effect was observed in anoxic stress model and cold restraint ulcer model. The exact reason for this difference in effect cannot be explained with the present data.

Clove is a known antioxidant and is reported to prevent nerve and vascular dysfunction in streptozotocin induced diabetic rats.[31] It is also known to scavenge free radicals generated during aflatoxicosis.[32] These effects are due to the presence of volatile oils, especially eugenol. Further, it is known that anti-oxidants can be beneficial for the prevention of stress induced pathological changes. The exact mechanism by which clove produces its anti-stress activity cannot be explained with the present data, however, it is speculated that the antioxidant effect of the clove buds might contribute at least in part to its anti-stress activity.
